# Implications of Childhood Autism Spectrum Disorder for Maternal Employment: United States vs. Norway

**DOI:** 10.1007/s10995-024-03961-z

**Published:** 2024-06-12

**Authors:** Idunn Brekke, Andreea Alecu, Celestia Ohrazda, Jiwon Lee

**Affiliations:** 1 Department of childhood and families, Division of Mental and Physical Health, Norwegian Institute of Public Health, PO Box 222, Skøyen, Oslo, N-0213 Norway; 2 Consumption Research Norway, Oslo Metropolitan University, Oslo, Norway; 3 Burton Blatt Institute, Syracuse University, New York, U.S.A.; 4 School of Nursing, Byrdine F. Lewis College of Nursing and Health Professions, Georgia State University, Atlanta, Georgia U.S.A.

**Keywords:** Autism Spectrum Disorder, Maternal Employment, Logistic Regression, Comparative Research

## Abstract

**Objectives:**

A country’s social welfare system may play an important role in maternal employment. This study compared the labor market participation of mothers of children within the United States (U.S.) and Norway to examine whether the child’s age and severity of the ASD affected mothers’ employment differently between the two countries.

**Methods:**

The 2019 National Survey of Children’s Health was used for the U.S. analysis, and the 2019 administrative register data were used for the Norwegian analysis. A logit model was used to analyze the impact of a child’s age and ASD severity on maternal employment in the U.S. and Norway. We presented the results as average marginal effects obtained from the logistic regression analyses.

**Results:**

After adjusting for mothers’ sociodemographic variables and the child’s age, U.S. mothers of children with mild ASD and moderate/severe ASD had respectively 12 and 25% points lower probability of being employed than U.S. mothers of children without special health care needs. In Norway, mothers of children with moderate/severe ASD had a 13% points lower probability of employment than mothers without special health care needs. The probability of being employed for mothers caring for a child with ASD was significantly greater as the child got older in both countries.

**Conclusions for Practice:**

The employment gap was more substantial in the U.S. than in Norway. A general high employment participation rate among women and an elaborated welfare state and policy package seem to benefit employment among mothers of children with ASD in Norway.

**Supplementary Information:**

The online version contains supplementary material available at 10.1007/s10995-024-03961-z.

## Significance

**What is Known about this Subject?:**

Previous research examining the relationship between ASD and maternal employment are dominated by small sample sizes and single-country design.

**What this Study Adds?:**

This study examine the implications of childhood ASD on maternal employment in two vastly different welfare regimes, U.S. and Norway using nationally representative large samples.

**Supplementary Information:**

The online version contains supplementary material available at 10.1007/s10995-024-03961-z.

## Introduction

Autism spectrum disorder (ASD) is a neurodevelopmental disability characterized by social and communication limitations, often accompanied by restricted and repetitive behaviors, interests, and activities (American Psychiatric Association (APA), 2013). While ASD prevalence estimate varies between countries (e.g.,16.8 per 1000 children in the United States [U.S.] vs. 7 per 1000 children in Norway) (Baio et al., 2018; Surén et al., 2019), in both countries the prevalence has increased over time (Zeidan et al., 2022). Children with ASD need support from multiple siloed agencies, such as educational and healthcare systems, and community-based resources (Hyman et al., 2020). Mothers are often the primary caregivers and face unique caregiving challenges in meeting educational, medical, and service needs, affecting mothers’ labor market participation (Lee, 2013). Previous studies reported that U.S. mothers of children with ASD were less likely to be employed and earned 56% less than the mothers of children without health limitations, simultaneously there were no significant impact on the fathers’ labor market participation (Cidav et al., 2012). Another U.S. study reported that parents of children with ASD reduced the number of work hours per week; however, the reduction was much smaller for fathers than for mothers (McCall & Starr, 2018). A study from Australia found that mothers of children with ASD had significantly greater odds of not participating in the labor force compared to mothers of children without ASD (Callander & Lindsay, 2018). A study from Sweden showed that parents of children with ASD were more likely to be on sick leave, not participating in the labor force, and have low earnings compared to parents who did not have a child with ASD (McEvilly et al., 2015). These associations were stronger among mothers than among fathers.

Previous research suggested that individual factors (e.g., child’s age, mother’s age, educational level, marital status, children having intellectual disabilities) and organizational factors (e.g., workplace policy, childcare availability, organizational culture) are associated with mothers’ labor force participation (Brown & Clark, 2017; Callander & Lindsay, 2018; Cidav et al., 2012; McCall & Starr, 2018). In addition, the welfare state regime plays an important role in maternal employment. Generally, there is a strong connection between the welfare state’s provisions and labor market participation (Vinck & Brekke, 2020). For example, family policies, including generous parental leave and state-sponsored childcare, facilitate the combination of care responsibilities with employment (Mandel & Semyonov, 2006).

This study compared labor market participation of mothers of children with ASD between two developed countries, the U.S. and Norway. Our study is unique because these two countries represent vastly different welfare systems. The Norwegian welfare state supports women’s participation in paid work institutionally and culturally and provides accessible quality childcare and generous parental leave schemes (Kuhnle & Pedersen, 2017). In comparison, the U.S. has a more market-oriented model leaving families to care for their children (Emmenegger et al., 2015). In addition, more women in Norway compared to in the U.S. are employed. The labor market participation rates among women are 66% in Norway and 57% in the U.S. (See Appendix Table A1). Appendix Table A1 presents an overview of the relevant family policy measures targeting children with special health care needs in the U.S. and Norway and additional information on the labor market conditions.

Understanding and assessing the effect of ASD on maternal employment would be a major contribution to implementing policies to support these families. Therefore, the purpose of this study is to: (1) examine the impact of ASD on maternal labor market participation in the U.S. and Norway and (2) examine whether any differences in employment participation between mothers of children with ASD and mothers of children without special health care needs depends on the age of the child and the severity of ASD. We expect that combining work with increased care responsibilities for a child with ASD may be less challenging for Norwegian mothers than U.S. mothers due to a more generous welfare state in Norway that aims to facilitate the combination of paid work and care responsibilities.

## Methods

For the U.S. data, we used data from the 2019 National Survey of Children’s Health (NSCH), a population-based survey based on a retrospective, cross-sectional design of non-institutionalized U.S. children aged 0 to 17 years regarding their health, quality of care, access to healthcare, family characteristics, and other factors affecting children’s health (The Child and Adolescent Health Measurement Initiative, 2022). For the norwegian data, we used the 2019 register data from Statistics Norway, specifically the Historical Event Database (FD-Trygd), the central population register linked to the National Education Database. This data contains all children born between 2000 and 2017 and their mothers.

### Sample

From NSCH 2019 data, children with ASD were identified by the parental response to the following question, “Has a doctor or health professional EVER told you that [subject child] has Autism or Autism Spectrum Disorder (ASD)? Include diagnoses of Asperger’s Disorder or Pervasive Developmental Disorder (PDD).” In order to better capture children with ASD, we only included parents who answered yes to the following question, “does this child CURRENTLY have the condition?”. The NSCH uses the children with special health care needs (CSHCN) screener questions to identify children with special health care needs. The CSHCN were identified based on five questions reflecting health consequences a child experiences due to an ongoing health condition (e.g., need or use of prescription medications, services, specialized therapies, functional difficulties, and emotional or behavioral problems for treatment). We defined children without ASD based upon the negative responses to the CSHCN screener questions. Our final U.S. sample included 456 children with ASD, and 10,316 children without special health care needs. Sampling weights were adjusted to account for non-response and to reduce the magnitude of bias. The survey responses were weighed (the survey response rate for 2019 was 42.4%), and details about the survey methodology and statistical weighting as well as missing data and imputation methods can be found at https://www.childhealthdata.org/learn-about-the-nsch/methods.

In the Norwegian register data, children with ASD were identified using the information about the diagnosis linked to assistance allowances obtained from the FD-Trygd and Norwegian Labour and Welfare Administration register (NLWA). Children who need long-term private care and supervision because of a medical condition are entitled to assistance allowances which is a non-mean tested state financial support provided by NLWA. Diagnostic codes are recorded in the NLWA register according to the World Health Organization’s International Classification of Diseases, version 10 (ICD-10). Children registered with ICD-10: F84, pervasive developmental disorders, including diagnoses of Asperger’s disorder and autism, were classified as children with ASD. Children who were not registered with assistance allowances were classified as children without special health care needs. Our final Norwegian sample included 1,531 children with ASD, and 346,363 children without health care needs. The survey parameters and weights for the Norwegian data were set to equal 1.

Children younger than 4 years were excluded from both samples because children under three are unlikely to have been diagnosed with ASD (McEvilly et al., 2015). This is particularly the case in Norway where we had very few children registered with ASD younger than 4 years old. Therefore, we narrowed the study population in the two countries to include only children 4 to 17 years old. Mothers in this study refer to biological or adoptive mothers.

### Variables

The dependent variable was mothers’ labor market participation. A single item was used to measure employment status. In the NSCH data, mothers were asked, “Were you [caregiver] employed at least 50 out of the past 52 weeks?” Responses included yes or no. Mothers are classified as employed if they respond yes to this question. In the Norwegian data, the mothers are classified as employed if they were registered as paid employees or were self-employed during the reference week (3rd week of November 2019) and had labor income of at least 1.5 (14,095 USD) of The Norwegian Social Insurance Scheme’s basic amount in 2019. The same threshold is used to qualified for unemployment benefit in Norway.

The main independent variable was an indicator of a child’s ASD. This variable was further stratified by the ASD symptom severity (mild, moderate, or severe) and children without ASD and special health care needs. The ASD symptom severity was identified by the parental response to the following question [“Would you describe [his/her] health condition[s] as mild, moderate, or severe?”]. In the NSCH data, 234 parents reported their child with ASD had mild ASD symptom severity, 222 parents reported their child with ASD had moderate or severe ASD symptom severity. In the Norwegian data, we used the level of assistance allowance payment as a proxy for ASD symptom severity, ranging from 1 to 4. We equated payment level 1 with a mild ASD (*n* = 82) and payment levels 2 to 4 with moderate or severe ASD symptom severity (*n* = 1,449).

We attempt to control for covariates related to the independent and outcome variables to make our assumption plausible. Child characteristics in our analyses included: the child’s age and gender. Mothers and household characteristics included: age, immigrant background, family structure, education, and household income level. In the NSCH data from the U.S., the household income was based on the Federal Poverty Level (FPL), divided into four income levels: 0-199%, 200–299%, 300–399%, and 400% or greater. In the Norwegian data, the household income (measured in Norwegian Krones) was divided into four groups: under 60% of median income, 60% of median-to-median income, median to 75%, and above 75%. Although the income variable is not entirely compatible in both countries, it is not a significant issue as it only serves as a control variable.

### Statistical Analysis

Statistical analyses were performed using STATA®17. For educational and income level variables in the NSCH data, the missing values were imputed per guidance from the Census Bureau using the NSCH. Descriptive analyses were presented with means, standard deviation (SD), and proportions (%). We compared variable means and proportions of children with mild and moderate/severe ASD symptom severity with children without special health care needs. The analyses of employment were performed using logistic regression to get insight into how maternal employment related to having a child with ASD. For that, we contrasted mothers caring for a child with ASD to a control group of mothers caring for a child without special health care needs using a combined dataset across the U.S. and Norway. We presented the results as average marginal effects obtained from the logistic regression analyses (Odd Ratio [OR] and 95% confidential interval [CI] can be found in Appendix Table A2. The marginal effects for the categorical variables indicate how the probability P (Y = 1) varies as the categorical independent variable changes from 0 to 1, holding all other independent variables in the analysis constant. For continuous independent variables, the marginal effect measures the instantaneous rate of change (Williams, 2012). Unlike ORs, marginal effects were not influenced by the amount of unexplained variance in the model and can be compared across models (Mood, 2009). We built three separate models, unadjusted estimates (model 1) and adjusted estimates after controlling for child, mother, and family characteristics (model 2) that included a full set of interactions between the independent and the country dummy variables (i.e., the U.S. and Norway). In addition, we examined if the two counties differed when comparing the relationships between the mothers’ employment and child’s age by three child’s groups (children without special health care needs vs. children with mild ASD vs. children with moderate/severe ASD) by including a three-way interaction term between child’s ASD status, child age and the country dummy variable (model 3). Since The Norwegian data are longitudinal; we performed an additional analysis to control for the mothers’ employment status before birth, (see Appendix Table A3). We used robust standard errors, and the statistical significance level was set to *p* < 0.05. The ethical and legal aspects of this study have been thoroughly evaluated. The Regional Committee for Medical Research Ethics in southeastern Norway approved this study (116,474).

## Results

### Sample Description

Table 1 presents sample characteristics by child ASD status in the U.S. and the Norwegian samples. U.S. mothers of children with moderate or severe ASD were less likely to have higher education or be in the highest income group (400% FPL or greater) than mothers of children without special health care needs. In addition, U.S. mothers of children with ASD were less likely to be married or cohabited than mothers of children without special health care needs. Comparable results were found in the Norwegian analysis. A higher share of mothers born outside Norway cared for children with mild ASD in Norway compared to the U.S. Other maternal demographic characteristics were similar between the U.S. and Norway.

**Table 1:** Descriptive statistics of variables used in the analysis by ASD status, in U.S. and Norway

	U.S.			Norway		
	Children with mild ASD (*n* = 234)	Children with moderate/severe ASD (*n* = 222)	Children without special health care needs (*n* = 10,316)	Children with mild ASD (*n* = 82)	Children with moderate/severe ASD (*n* = 1.449)	Children without special health care needs (*n* = 347,894)
**Child characteristics**						
Age in years, mean (SD)	11.79 (3.75)	11.64 (4.01)	10.81 (4.19)	10.04 (4.59)	11.02 (4.02)	10.44 (3.9)
Boys,%	82.08	81.08	49.43	77.78	81.11	50.76
Girls, %	17.95	18.92	50.57	22.22	18.89	49.23
**Mother characteristics**						
Age in years, mean (SD)	42.82 (7.12)	42.62 (8.45)	41.70 (7.28)	39.11 (6.55)	39.33 (6.23)	38.47 (6.30)
Born in USA /Norway, %	93.16	89.19	86.75	46.67	60.70	72.24
Born outside USA/Norway, %	6.84	10.81	13.25	53.33	39.30	29.95
**Highest completed education**						
Compulsory education, %	1.28	7.66	3.72	15.51	23.17	19.05
Upper secondary or high school or less, %	45.30	53.15	42.25	28.05	31.43	26.90
Bachelor or higher, %	53.42	39.19	54.03	48.78	49.52	57.58
**Marital status**						
Married or cohabiting, %	76.50	72.97	81.75	88.89	85.83	90.51
Not married or cohabiting	23.50	27.03	18.25	11.11	14.17	9.49
**Household characteristics**						
**Number of children in the household, %**						
Single child	39.48	45.30	39.48	28.89	20.47	15.17
Two children	38.98	35.90	38.98	42.22	48.19	50.21
Three children	14.55	14.10	14.55	20.00	21.00	24.56
Four or more children	6.99	4.70	6.99	8.89	10.34	10.07
Income level of child’s household*, %						
Lowest income group	37.18	45.95	28.70	26.67	22.64	18.71
Income group 2	16.67	15.77	16.75	46.67	39.21	31.90
Income group 3	13.25	15.77	16.58	13.33	21.32	25.20
Highest income group	32.92	22.52	37.97	13.33	16.83	24.19
**Maternal employment**						
Employed %	64.53	49.55	75.59	67.09	60.74	79.55

Note: Data are mean (SD) or n (%), *In the USA data: lowest income group = 0-199% FPL, income group 2 = 200–299% FPL, income group 3 = 300–399 FPL, highest income group = 400% FPL or greater. In the Norwegian data: lowest income group = > 60% of median income, income group 2 = 60% of median income to median, income group 3 = median to 75% of median income, highest income group = above 75% of median income

### Maternal Employment

Compared to Norwegian mothers, U.S. mothers were less likely to report being employed. In the U.S. sample, 65% of children with mild ASD, 50% of children with moderate or severe ASD, and 76% of the children without special health care needs had working mothers. The comparable numbers for the Norwegian sample were: 67%, 61%, and 80%, respectively (Table 1).

**Fig. 1:**
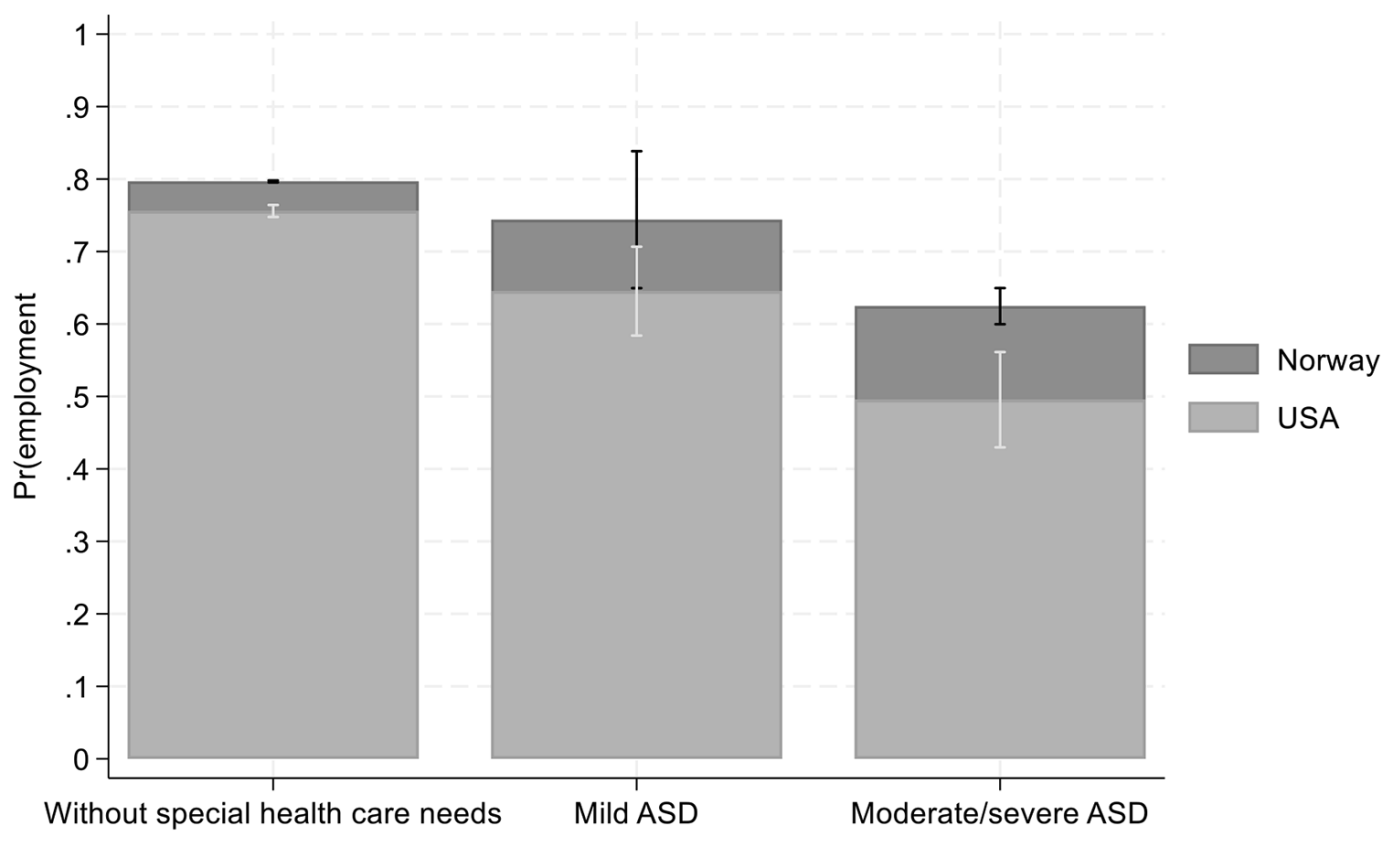
Dependent variable: maternal employment. Average marginal effects (AME) obtained from unadjusted logit regression coefficients, mothers of children with ASD and mothers of children without special health care needs in U.S. (*n* = 10, 772) and Norway (*n* = 347,894)

**Fig. 2:**
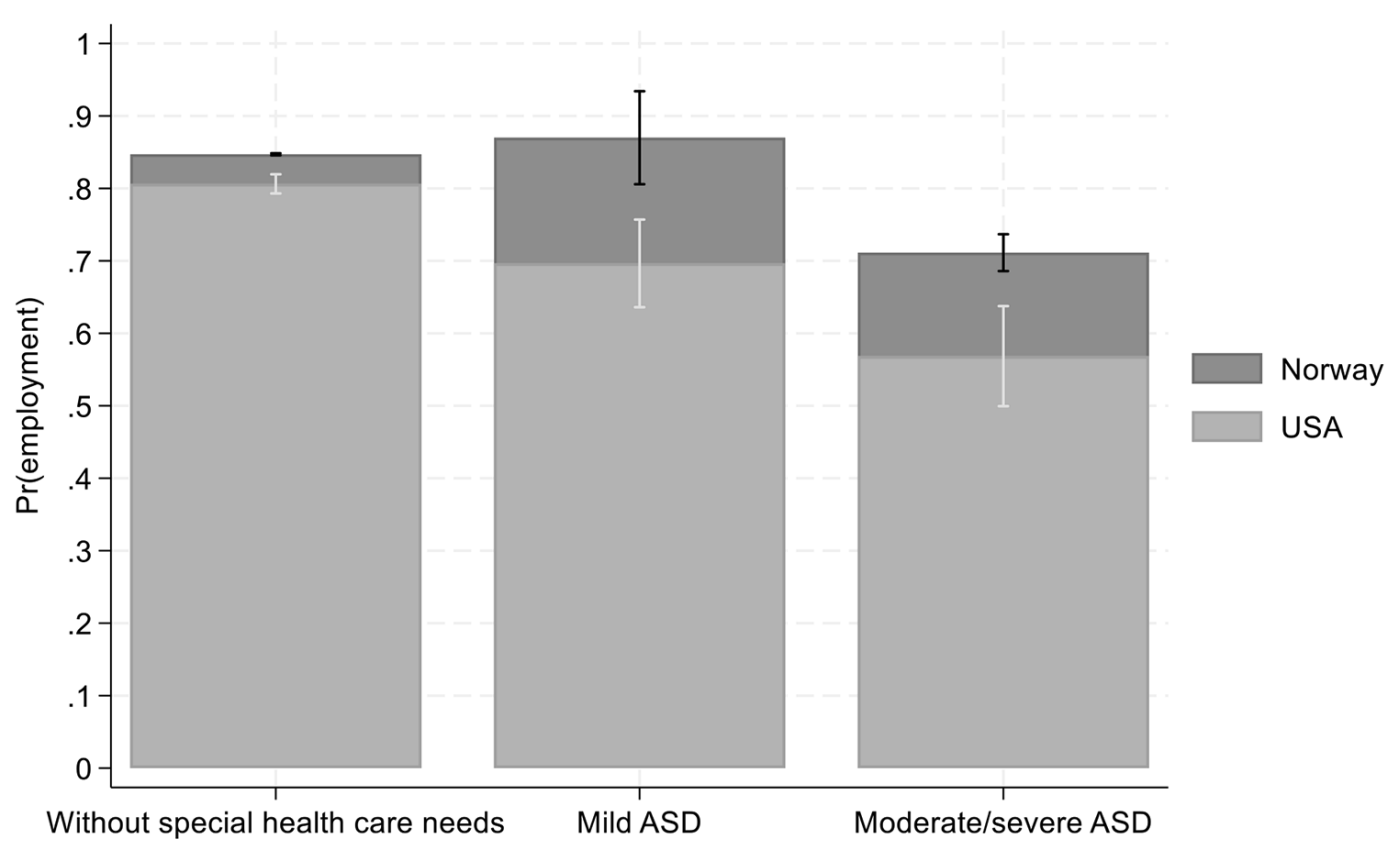
Dependent variable: maternal employment. Average marginal effects (AME) obtained from adjusted logit regression coefficients, mothers of children with ASD and mothers of children without special health care needs in U.S. (*n* = 10, 772) and Norway (*n* = 347,894) Note: the coefficients are adjusted for country, child age, child sex, mothers age, educational level, immigrant status, marital status, numbers of children in the household and household income, and a full set of interactions between the independent and the country dummy variables

**Fig. 3:**
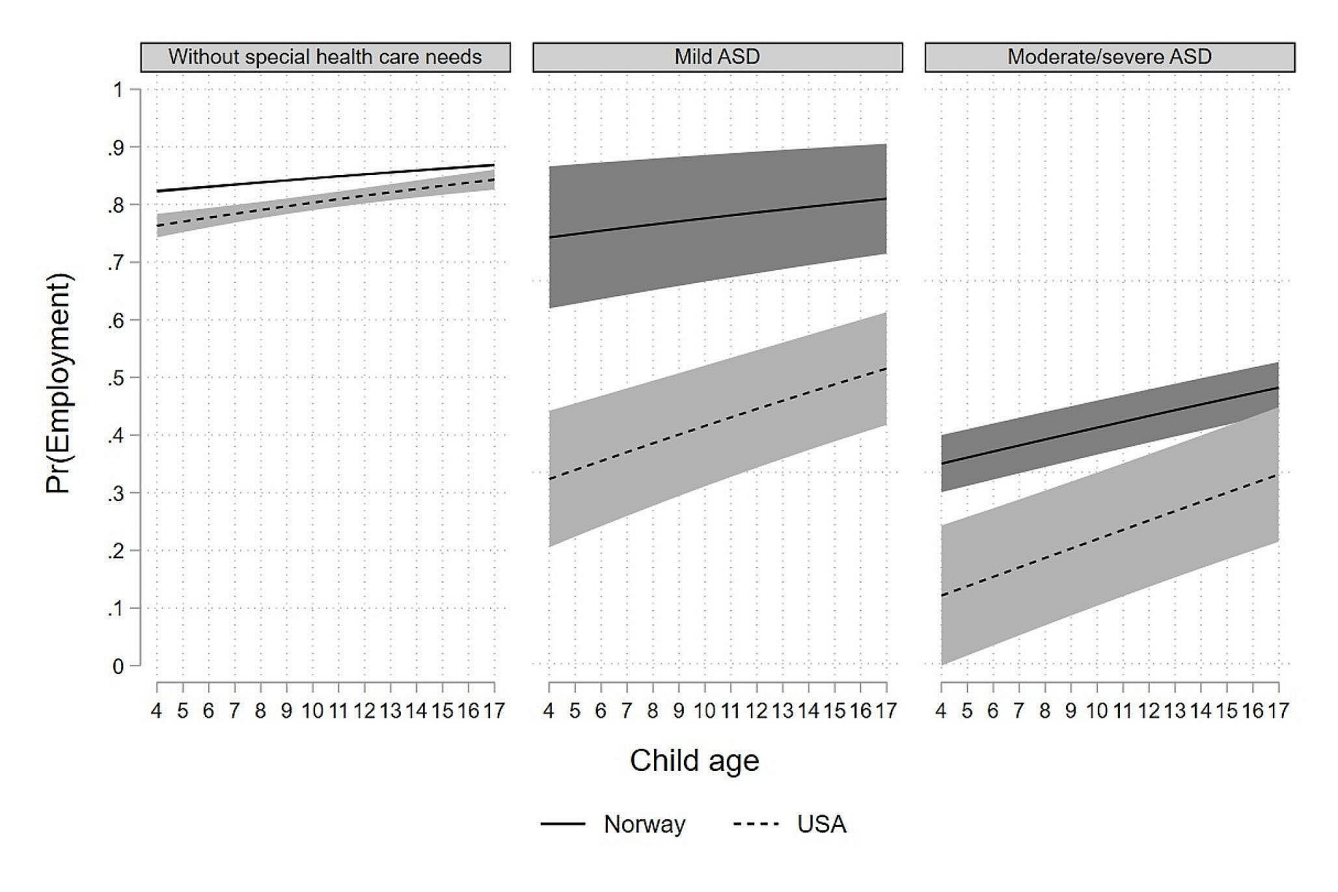
Average marginal effects (AME) of having a child with ASD on maternal employment in U.S. and Norway by child age, mothers of children with ASD and mothers of children without special health care needs in U.S. (*n* = 10, 772) and Norway (*n* = 347,894) Note: the coefficients are adjusted for country, child age, child sex, mothers age, educational level, immigrant status, marital status, numbers of children in the household and household income, and a three-way interaction term between child’s ASD status, child age and the country dummy variable

The multivariate analyses investigated whether there were significant differences in employment, among mothers caring for a child with ASD and mothers caring for children without special health care needs in U.S. and Norway. The probability of being employed are shown in Figs. 1, 2 and 3 as average marginal effects (AME). ORs and 95% CI are reported in Appendix Table A2. The relationship between ASD status and maternal employment is presented in three separate models. The first model presented the estimates without controlling for potential confounders (Fig. 1). The second model controlled for children’s, mothers, and family characteristics (Fig. 2). The third model tested if child’s age moderated the relationship between ASD status and maternal employment in U.S and Norway (Fig. 3). U.S. mothers of children with mild ASD and moderate/severe ASD had 11 and 26% points lower probability of being employed than mothers of children without special health care needs. Norwegian mothers of children with moderate or severe ASD had a 17% points lower probability of being employed than mothers of children without special health care needs. There were no significant employment differences between mothers of children with mild ASD and mothers of children without special health care needs in Norway.

After adjusting for mothers’ socio-demographic variables and the child’s age (Fig. 2), mothers of children with ASD in both countries had lower probability of being employed than mothers of a child without special health care needs. U.S. mothers of children with mild ASD had 12% points lower employment probability compared to mothers of children without special health care needs. The corresponding numbers for U.S. mothers caring for children with moderate/severe ASD was 25% points. Norwegian mothers of children with moderate/severe ASD had 13% points lower employment probability compared to mothers of children without special health care needs. In the additional Norwegian analysis where we control for the mothers’ employment status before birth (see Appendix Table A3) comparable results are obtained.

Overall the result demonstrated that the employment gap was significantly more substantial in the U.S. compared to Norway in both the unadjusted and the adjusted analysis.

Moreover, the results showed that the probability of the mother of children with mild and moderate/severe ASD being employed was significantly greater as the child gets older in both U.S. and Norway (illustrated in Fig. 3). The employment gap between mothers of children with ASD and mothers of children without special health care needs decreased as a child gets older. However, this pattern is more pronounced in U.S. than in Norway, as we see that the lines are steeper in U.S. than in Norway. Moreover, the figure shows that the probability of being employed for Norwegian mothers of children without special health care needs almost stays flat, while it increases for U.S. mothers.

## Discussion

We examined whether maternal employment differs between mothers who have a child with ASD and those without, in both the U.S. and Norway. We also explored how this employment gap varies depending on the child’s ASD severity and age. To our knowledge, this is one of the first studies to compare mothers of children with ASD’s employment between the two counties, using comparable data sets.

The present study found that mothers of children with ASD were less likely to be employed than mothers of children without special health care needs. While we cannot be certain that the reduced employment we observed is caused by having a child with ASD, the results align with previous research showing that mothers of children with ASD are at substantial risk of reduced employment (Callander & Lindsay, 2018; Cidav et al., 2012; McCall & Starr, 2018; McEvilly et al., 2015). Thus, it is reasonable to suggest that caring for a child with ASD negatively impacts a mother’s employment.

As we expected, the employment gap was more pronounced in the U.S. than in Norway. The reason for the difference between the U.S. and Norway could be attributed to various systematic and individual factors. While Norwegian legislation gives every child over 12 months old the individual right to affordable, high-quality childcare, many parents, particularly those who care for a child with a disability, struggle to find high-quality, affordable care for their child in the U.S. (Costanzo & Magnuson, 2019). Although coverage for the diagnosis and treatment of ASD is mandated via private and public health insurance (Autism Speaks, 2020), there is a gap in ASD-related services in the U.S. For example, access to Board Certified Behavior Analysts (BCBAs) for applied behaviour analysis (ABA) services in children with ASD differed by counties sociodemographic factors, with affluent and urban counties more likely to have greater access to the BCBAs (Yingling et al., 2021). Another study highlighted that U.S. parents faced great challenges in securing proper services for their children with ASD (Helkkula et al., 2020). Norway’s universal health care services are provided free for all children 16 years and younger (Özerk & Cardinal, 2020). These factors suggest that the differences in the employment gap in U.S. and Norway might come from differences in welfare state systems and policy factors.

While the negative relationship between mothers’ employment and having a child with ASD applied to mothers in both countries, our study demonstrated that the employment gap was more significant among mothers of children with moderate or severe ASD than among mothers of children with mild ASD. Our results are in accordance with a systematic review showing that the severity of the child’s disability has been linked to lower levels of work-family balance (Brown & Clark, 2017). Children with a more severe ASD require a great deal of caregiving. Balancing between care and employment may be particularly challenging for these mothers. However, we found no significant differences between mothers of children with mild ASD and mothers of children without special health care needs in Norway. This result must be understood in the context that the group with mild ASD was small and that they were only administratively recognized if they received assistance allowance. The result must therefore be interpreted with caution.

The present study also showed that maternal employment varied according to the child’s age in both countries, with significantly greater maternal employment as the child ages. This applied to all mothers, irrespective of the child’s ASD status, except for Norwegian mothers of children without special health care needs; these mothers did not have significantly increased participation in the labor market as the child aged (measured from 4 years of age). An explanation could be that Norwegian mothers initially have a high employment rate when the child has turned four years old, and therefore it is unlikely to see increases in the employment rate.

Moreover, the study shows that the difference in employment probabilities between the mother of children with ASD and mother of children without special health care needs decreases as the child ages in both countries. This pattern is particularly pronounced in the U.S. Possible reasons explaining decreased employment gaps between mothers of children with ASD and mothers of children without special health care needs may be that the first year of ASD diagnosis may be the most critical, possibly due to major adjustments that must be made when the mothers return to work and the child begins childcare. The main conclusion from this study is that having a child with ASD has a substantially negative impact on maternal employment, particularly in the U.S. Lower maternal employment has a negative implication for families of children with ASD’s financial well-being as U.S. mothers of children with ASD earned 35% less than the mothers of children without health limitations and were more likely to work part-time (Cidav et al., 2012). Working U.S. mothers of school-aged (6–17 years) children with disabilities reported better mental health compared to their non-working counterparts, indicating a beneficial influence of employment on mothers’ mental health (Morris, 2014). Comparable findings were observed in a European study (Tokić et al., 2023). Workplace policy and supervisory support positively impacted employment and retention in parents of children with disabilities (Brown & Clark, 2017). Cultivating workplace culture to protect and encourage working mothers to request alternative or flexible work schedule may be helpful for U.S. mothers of children with ASD to increase paid work participation. One study reported most mothers of children with developmental disabilities were unaware of their right to request a change in working arrangements (Crettenden et al., 2014). Increased awareness of legislation and flexible leave entitlements among U.S. mothers of children with ASD is another necessary step.

The strengths of this study include that this study was the first comparable study between the U.S. and Norway using a comparable dataset and used the large sample size, with a wide range of sociodemographic and health variables. Another strength of this study is the inclusion of data on children with different ASD severity level. Thus, it was possible to analyze differences in maternal employment according to the child’s severity level.

We also want to acknowledge limitations of this study. First, the causal relationship between a child’s ASD status and maternal employment cannot be drawn, given that this study was based on cross-sectional data. Mothers may have unobserved characteristics affecting their employment. The Norwegian data are longitudinal; thus, we can control for the mothers’ employment status before birth (see Appendix Table A3). These analyses show comparable results, which strengthens the robustness of our results. Second, in the Norwegian data, we only considered children with ASD if they were administratively recognized and received assistance allowances; thus, we may not capture less severe ASD conditions. However, we controlled for ASD severity level in the analysis in both countries, which minimized this problem. The diagnoses recorded in the Norwegian data used in this study are restricted to the main diagnoses. Some of the children included in this study may have various combinations and degrees of comorbidities that we cannot account for in our analysis. Finally, the definition of the employment variable is somewhat stricter in U.S. than in Norway. However, we use additional information on labour income in the Norwegian data to make the employment variable more comparable across the two countries.

Despite these limitations, our findings have important policy implications. Families raising children with ASD are more likely to face low maternal employment. However, the context of the country where the mother works and lives is important. The employment gap is smaller in Norway compared to the U.S. A general high employment participation rate among women and an elaborated welfare state and policy package seem to benefit employment among mothers of children with ASD in Norway. Further research is necessary to pinpoint the specific individual and systematic factors that impact employment opportunities and barriers for mothers of children with ASD in the U.S.

## Electronic Supplementary Material

Below is the link to the electronic supplementary material.

Supplementary Material 1

## Data Availability

The Norwegian data used in this study are available from Statistic Norway, but they are not publicly accessible because they were used under license for this study. The data may, however, be made available upon request to the first author granted permission is given by Statistic Norway. The data used in this study from U.S. are available from The Data Resource Center for child and adolecnt Health.

## Code Availability

The syntax could be made available upon request to the first author.
