# Supplementary material for: Implications of Childhood Autism Spectrum Disorder for Maternal Employment: United States vs. Norway
Source: Matern Child Health J. 2024 Jun 12;28(10):1707–15. doi: 10.1007/s10995-024-03961-z (PMC11420245; doi:10.1007/s10995-024-03961-z)
Supplement: Supplementary file 1 — Supplementary Material 1 [file 10995_2024_3961_MOESM1_ESM.docx]

**Supplementary appendix:**

TABLE A1. Overview of U.S. and Norwegian policies for children with special health care needs and labor market information, 2019

|  | **U.S.** | **Norway** |
| --- | --- | --- |
| **Government benefits** | - Supplemental Security Disability Income (SSDI): Household income based - Medicaid: Household income based - Medicaid Waiver: Waives some Medicaid requirements (such as income). Differs from state to state - Children’s Health Insurance Program (CHIP): Uninsured children in families with incomes too high to qualify for Medicaid, but too low to afford private coverage | ***Assistance allowance***  • No age limit  • Not income-based  • Norwegian Labor and Welfare Administration (NLWA) recognition needed  • Severity-adjusted  ***Basic benefit***  • No age limit  • To cover additional expenses related to medical conditions (excluding medication)  • NLWA recognition needed  • Adjusted to severity of medical conditions |
| **Child care/ educational services** | ***The Family and Medical Leave Act (FMLA)***  ***Early Intervention*** (0 - 3 years)   - Early childhood diagnosis - Therapy and services in the home and daycare - Special education daycare services   ***Individualized Education Programs (IEP)*** (3–21 years)   - Outlines the services the school will provide to facilitate child’s learning goals | ***Integrated childcare***  • Integrated into regular childcare system  • Prioritization of children with increased care needs over other children  ***Other care services***  • Municipal competence  • Duty to organize coordination units  • Municipal NLWA recognition needed  • Support personnel, relief and personal assistance |
|  | **U.S. (%)** | **Norway (%)** |
| **Employment rate, women** | 57.4 | 65.6 |
| **Unemployment rate, women** | 3.6 | 3.4 |

Note: ***FMLA*** is eligible employees of covered employers to take unpaid, job-protected leave for specified family and medical reasons with continuation of group health insurance coverage under the same terms and conditions as if the employee had not taken leave. https://www.dol.gov/agencies/whd/fmla. ***Early intervention*** is an early intervention services for children with disabilities. IEP is a legal document aim to optimize educational services for children with special health care needs and it is only applied for public school system. ***Assistance allowance*** is a non-means-tested cash benefit adjusted to the severity of increased care needs for which parents need to file an application. The care needs must last for two to three years or more. The benefit is paid at four different rates, reflecting mild to severe care needs. The overall workload of the person providing the care/supervision is the determining factor. https://www.nav.no/hjelpestonad/en. Employment information for Norway can be found: <https://www.ssb.no/arbeid-og-lonn/artikler-og-publikasjoner/flere-heltidssysselsatte-kvinner>. For U.S.: https://www.bls.gov/opub/reports/womens-databook/2020/home.htm#:~:text=the%20technical%20notes.)-,Selected%20demographic%20characteristics,previous%20year%20(69.1%20percent).

TABLE A2. Dependent variable: maternal employment. OR and 95 % CI for U.S., (n=10,772) and Norway (n=347,894)

|  | **Model 1 (unadjusted)** | | **Model 2**  **(adjusted)** | |
| --- | --- | --- | --- | --- |
|  | **OR** | **95 % CI** | **OR** | **95 % CI** |
| **Country** |  |  |  |  |
| Norway (ref) |  |  |  |  |
| USA | 0.79 | 0.75-0.82 | 4.68 | 3.19-6.86 |
| **ASD status** |  |  |  |  |
| Without special health care needs (ref) |  |  |  |  |
| ASD mild | 0.74 | 0.45-1.21 | 1.20 | 0.65-2.12 |
| ASD moderate/severe | 0.42 | 0.38-0.47 | 0.44 | 0.39-0.50 |
| **ASD status X country** |  |  |  |  |
| ASD mild X USA | 0.79 | 0.45-1.39 | 0.45 | 0.24-0.86 |
| ASD moderate/severe X USA | 0.74 | 0.56-0.99 | 0.71 | 0.52-0.96 |
| Constant | 3.91 | 3.88-3.95 | 0.30 | 0.28-0.32 |

Reference categories: “Norway”, ‘without special health care needs’; Model 1: unadjusted results. In model 2, the coefficients are adjusted for child age, child sex, mothers age, educational level, immigrant status, marital status, numbers of children in the household and household income, and a full set of interactions between the independent and the country dummy variables.

TABLE A3. Dependent variable: employment. Average marginal effects (AME) obtained from logit regression coefficients, mothers of children with ASD and mothers of children without special health care needs in Norway adjusted for employment status prior to birth

| **Norway** | | | | | | |
| --- | --- | --- | --- | --- | --- | --- |
|  | **Model 1** | | | **Model 2** | | |
|  | **AME** | **SE** | **P-value** | **AME** | **SE** | **P-value** |
| **ASD Severity** |  |  |  |  |  |  |
| ASD mild | -0.05 | 0.04 | 0.22 | -0.01 | 0.03 | 0.76 |
| ASD moderate/severe | -0.15 | 0.01 | 0.000 | -0.90 | 0.01 | 0.000 |
|  |  |  |  |  |  |  |

Reference categories: ‘without special health care needs’; Model 1: unadjusted results. In model 2, the coefficients are adjusted for child age, child age squared, child sex, mothers employment the year status prior to birth, mothers age, mothers age squared, educational level, immigrant status, marital status, numbers of children in the household and household income.
